# A Novel, Modified Reverse Controlled Antegrade and Retrograde Subintimal Tracking Technique for Bypassing the Calcified Proximal Cap of Coronary Total Occlusions

**DOI:** 10.1155/2017/3850646

**Published:** 2017-04-26

**Authors:** Tsuyoshi Isawa, Masahiko Ochiai, Masato Munehisa, Tatsushi Ootomo

**Affiliations:** ^1^Department of Cardiology, Sendai Kousei Hospital, Sendai, Japan; ^2^Department of Cardiology and Cardiovascular Surgery, Showa University Northern Yokohama Hospital, Yokohama, Japan

## Abstract

Antegrade crossing is the most common approach to chronic total occlusions (CTOs). However, it is sometimes difficult to penetrate the proximal hard cap with guidewires, especially in the case of CTOs of anomalous coronary arteries because of a lack of support. Herein, we describe a novel, modified reverse controlled antegrade and retrograde subintimal tracking (CART) technique in which the dissection reentry was intentionally created in the proximal segment of the vessel, not within the occluded segment, using retrograde guidewire and the aid of an antegrade balloon. This technique facilitated retrograde crossing of CTOs by avoiding the proximal hard cap and may provide a viable option for patients in which conventional reverse CART is not possible.

## 1. Introduction

A chronic total coronary occlusion (CTO) is defined as the complete obstruction of a coronary artery that is more than 3 months old [1]. The recent meta-analysis revealed that successful recanalization of a CTO resulted in the overall improvement of left ventricular ejection fraction, reduced adverse remodeling, and an improvement of survival [2]. In percutaneous coronary intervention (PCI) for CTO, antegrade crossing is commonly used to target CTO lesions [3]. However, penetrating the proximal hard cap with guidewires is challenging, especially in cases of anomalous coronary arteries. Consequently, contemporary reverse controlled antegrade and retrograde subintimal tracking (CART) [4], one of the most common techniques used in retrograde PCI, cannot be applied in such cases due to failure in antegrade preparation (creating an antegrade dissected space using an antegrade wire and balloon). To overcome such obstacles, we herein present a novel, modified reverse CART technique in which the dissection reentry is intentionally created in the proximal vessel, not within the occluded segment, by an antegrade balloon, which is followed by maneuvering the retrograde guidewire through the subintimal space into the proximal true lumen.

## 2. Case Presentation

**Figure 1:**
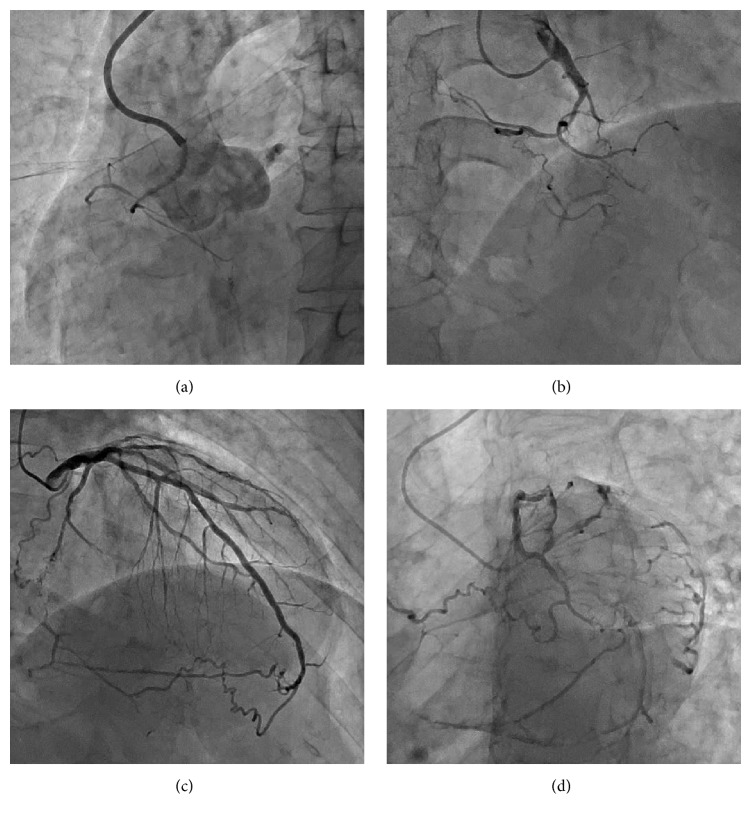
Profile of chronic total occlusion (CTO) of the patient. (a) CTO of the proximal right coronary artery (RCA) (right anterior oblique projection). Coronary angiography revealed an anomalous origin of RCA with high anterior take-off from the aorta. (b) CTO of the proximal RCA (left anterior oblique projection). Epicardial collateral channels via the apex from the left anterior descending artery to RCA [right anterior oblique projection (c) and spider projection (d)].

A 65-year-old man with hypertension and diabetes mellitus presented to our hospital with Canadian Cardiovascular Society Class III angina despite being on optimal medical therapy, including beta-blockers and long-acting nitrates. An electrocardiogram revealed normal sinus rhythm at a rate of 66 beats/min without abnormal QS wave patterns. An echocardiogram revealed normal overall left ventricular function. The diagnostic angiography showed a CTO in the mid-segment of the anomalous right coronary artery (RCA) with a high anterior take-off from the ascending aorta and a proximal cap ambiguity of RCA-CTO because of side branches, including the sinoatrial node artery and right ventricular artery, arising near the occlusion site. The Japan-CTO score, a 5-point scoring system to assess the difficulty of CTO crossing [5], was 3. The distal vessel received several tortuous epicardial collateral channels between the left anterior descending artery (LAD) and posterior descending artery via the apex. To note, there were no interventional collateral channels from the septal arteries to the distal RCA (Figure 1).

**Figure 2:**
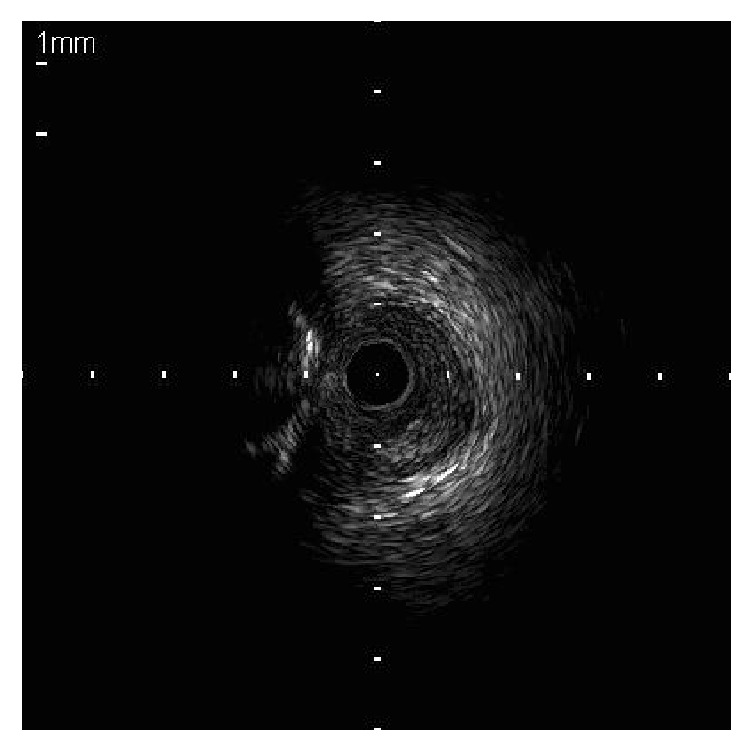
The calcified plaque blocks the entry of chronic total occlusion.

After discussion among the clinical heart team, PCI of a RCA-CTO was scheduled. Vascular access was obtained using bilateral femoral 7-Fr and 8-Fr long sheaths. Particularly, 7-Fr Hyperion AL 1 (Asahi Intecc, Aichi, Japan) and 7-Fr CLS 3.5 (Boston Scientific, Natick, MA, USA) with side holes were utilized to engage RCA and the left main coronary artery, respectively. Intravascular ultrasound (IVUS) examination from the right ventricular artery revealed that the heavily calcified plaque blocked entry of the CTO, suggesting difficulty in any guidewires penetrating into the CTO lesion (Figure 2).

**Figure 3:**
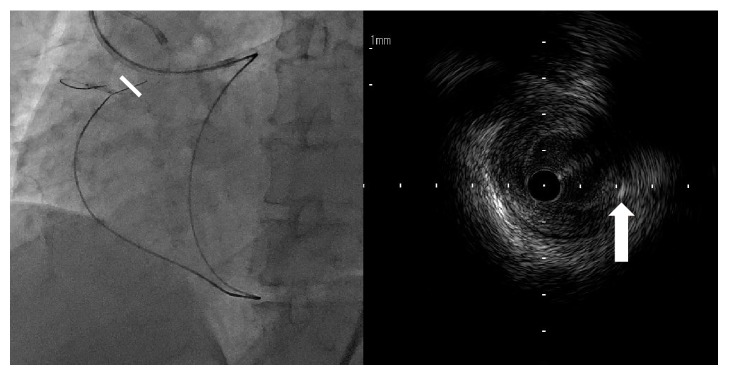
The intravascular ultrasound revealed that the retrograde guidewire (white arrow) was in the subintimal space at the proximal right coronary artery past the chronic total occlusion.

**Figure 4:**
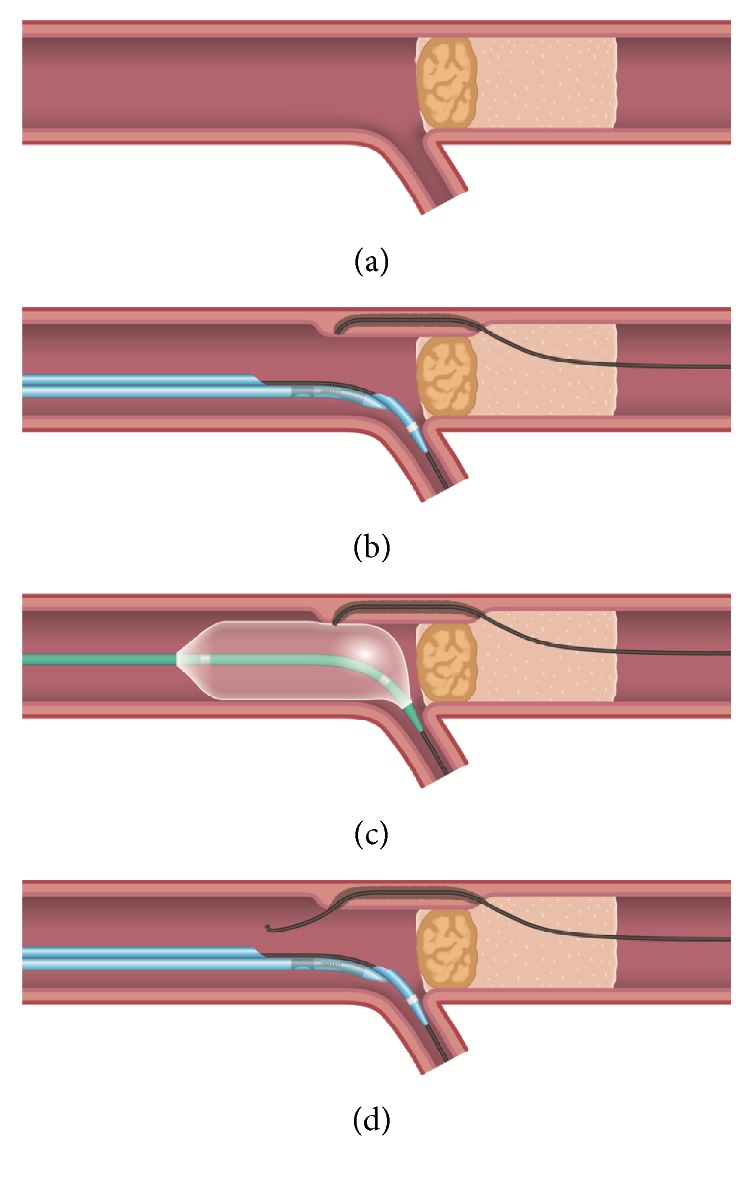
(a) A chronic total occlusion (CTO) in the right coronary artery. (b) A Gaia Third guidewire (Asahi Intecc) is retrogradely advanced into the subintimal space past the CTO ostium. (c) An antegrade balloon-assisted subintimal reentry is performed, which is created proximally to the occluded segment using a large balloon. (d) The retrograde guidewire successfully penetrated the proximal true lumen.

**Figure 5:**
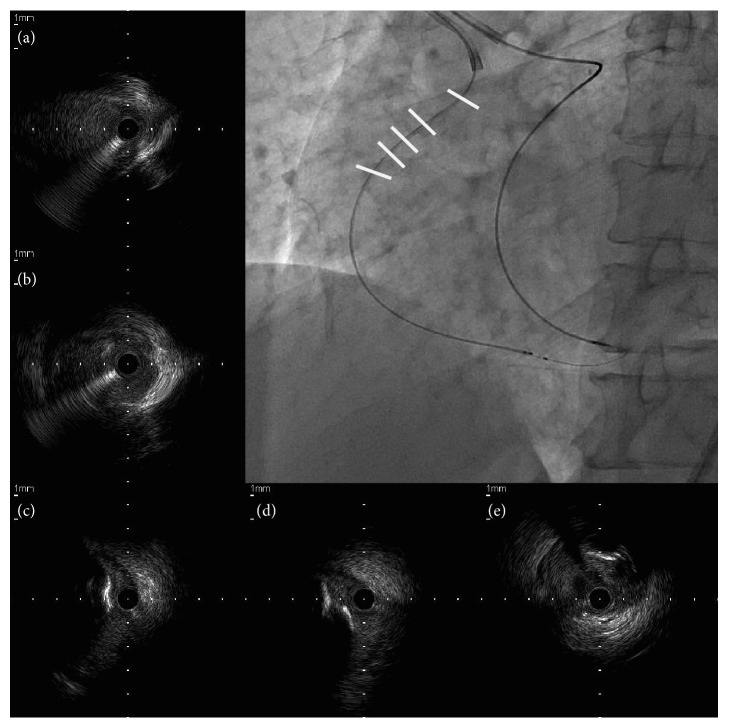
Intravascular ultrasound (IVUS) examination. IVUS was performed after guidewire externalization. Serial IVUS images of right coronary artery (RCA) show the ostial RCA (a), the guidewire entering into the true lumen from the false lumen at the proximal RCA (b), and the guidewire passing the chronic total occlusion (CTO) segment through the subintimal space, avoiding the severely calcified plaque at the proximal segment of CTO ((c)–(e)). Images are viewed in a proximal (a) to distal (e) direction.

**Figure 6:**
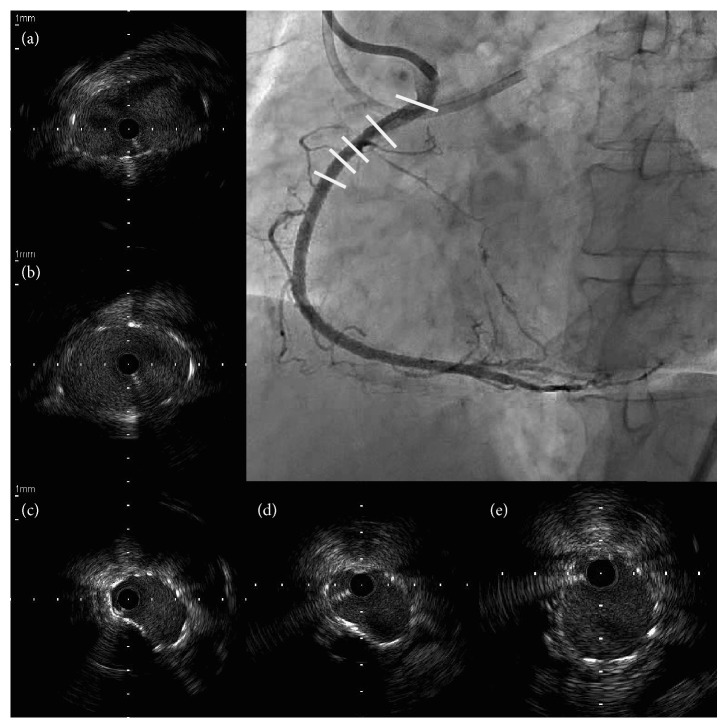
A final angiogram reveals an acceptable outcome. Serial intravascular ultrasound images of right coronary artery (RCA) after stenting show the ostial RCA (a), RCA proximal to chronic total occlusion (CTO) (b), and the CTO segments ((c)–(e)). Images are viewed in a proximal (a) to distal (e) direction. Adequate stent expansion is achieved throughout the lesion, except elliptical stent expansion limited by a superficial calcified lesion at the calcified proximal cap of CTO (c).

Therefore, a primary retrograde strategy using the epicardial collateral channels via the apex was performed. A Corsair Pro (Asahi Intecc) catheter was advanced into the distal LAD over a workhorse wire. Tip contrast injection revealed epicardial collateral channels. A SION guidewire (Asahi Intecc) was used as a first-line guidewire to cross the collateral channels, but it was unable to be crossed. Instead, for smoother and softer channel navigation, a SUOH 03 guidewire (Asahi Intecc) was used, which successfully traversed the collateral channels, while forming a knuckle, and thereafter a Corsair Pro catheter could follow (Movies 1 and 2 in Supplementary Material available online at https://doi.org/10.1155/2017/3850646). Reaching the distal end of CTO, the SUOH 03 guidewire was exchanged for a Gaia Third guidewire (Asahi Intecc). It was advanced retrogradely into the subintimal space past CTO ostium (Figure 3). Then, an antegrade balloon-assisted communication with the subintimal space was created proximally to the occluded segment using a 4.0 × 15.0 mm trek (Abbott Vascular), where the retrograde Gaia Third guidewire was entered, successfully penetrating the proximal true lumen (Figure 4) and reaching the aorta in a retrograde manner (Movie 3). The Corsair Pro was advanced similarly, followed by retrograde guidewire externalization with an RG3 300 cm guidewire (Asahi Intecc). The IVUS showed that the externalized guidewire traversed the CTO lesion, avoiding the heavily calcified plaque of the ostium (Figure 5). Afterwards, four drug-eluting stents were sequentially deployed from the distal to proximal RCA, and the final angiogram provided assurance, thereby allowing the procedure to be completed without any complications (Figure 6). The patient is currently free of symptoms after a 1-month follow-up.

## 3. Discussion

In this case report, we described a novel modified reverse CART technique for impenetrable CTOs in combination of epicardial collateral channels crossing using a SUOH 03 guidewire. The present case revealed that the novel modified reverse CART was feasible in CTO lesions with severely calcified proximal cap, even if an antegrade guidewire could not advance into the CTO segment, by retrogradely advancing a controllable guidewire into subintimal space past the CTO ostium, with a retrograde guidewire bypassing the calcified proximal cap of CTO.

In this novel technique, dissection was intentionally created in the proximal vessel, not within the occluded segment, using an antegrade balloon inflation, and reentry was achieved in the proximal vessel, using a retrograde guidewire. Strictly speaking, we did not check if the antegrade balloon actually caused intimal disruption with intravascular ultrasound. However, a balloon that was large enough to cause intimal disruption was chosen, although the balloon-to-artery ratio should always be less than 1.19 for preventing coronary perforation [6].

In the contemporary reverse CART technique, the connection is established within the occluded segment to minimize the length of subintimal stenting and prevent side branch loss [4]. However, reverse CART technique requires antegrade preparation, where a small antegrade balloon is inflated near the distal end of CTO, before retrograde wiring [4]. On the other hand, the novel technique we described here can be applied to impenetrable and suspected impenetrable CTOs even if antegrade preparation is impossible or unsuccessful. In comparison with the previous report [7], in this study, dissection is created proximal to the CTO and reentry is also achieved in the true lumen proximal to the CTO. The method presented here is included in the “Hybrid Approach” in PCI of the CTO lesions. In the Hybrid Approach, retrograde intentional dissection is very frequently used to get around heavily calcified CTO segments. Our novel method has several advantages. First, it can be applied to any CTO lesions where strong backup support by the antegrade guiding catheters is difficult to obtain. Entering into the proximal cap of CTO requires enough backup force, which would have been difficult in our patient with anomalous RCA-CTO without any side branches suitable for the anchor balloon technique. On the other hand, entering into the distal occluded segment by retrograde guidewire is easier because the distal cap of the CTO lesions is usually softer than the proximal cap [8]. Another advantage of our technique is that proximal reentry into the true lumen is easier than distal reentry because of the larger caliber vessel. Lastly, the availability of IVUS, if suitable side branch is present, can help operators confirm the position of the retrograde guidewire within the vessels.

A new specific guidewire, SUOH 03, is of great use in crossing epicardial collateral channels, which is one of the most important steps in reverse CART. Septal collateral channels are safer than the epicardial ones and should be preferred in most cases [9]. However, we have no choice but to use epicardial collateral channels in cases where the septal connections cannot be visualized. Epicardial channels are challenging for generally recommended guidewires, such as SION and Fielder XTR (Asahi Intecc). We have demonstrated that introduction of the SUOH 03 guidewire allowed for much smoother and softer channel navigation. It is extremely flexible from the tip; the tip load is only 0.3 gram-force and therefore enables crossing the small bended vessels, as shown in the present case.

There are a few limitations to this modified reverse CART technique. It cannot be applied to occluded vessels that have significant proximal side branches because subintimal reentry and subsequent stent deployment can cause side branch loss. Establishing retrograde access through collateral channels is mandatory for application of this technique but is not always successful. In addition, diffuse and very heavy calcification within CTO, which disturbs direct retrograde crossing or knuckle wiring, may prevent use of this technique.

## 4. Conclusions

In summary, a modified, reverse CART technique combined with a retrograde approach for bypassing the calcified proximal cap was effectively and safely utilized in our patient. Successful passage of tortuous collateral channels, which is an important step of this technique, was accomplished by the use of a new specific guidewire, SUOH 03.

## Supplementary Material

Movies 1 and 2. A SUOH 03 guidewire successfully crossing the collaterals while forming a knuckle, followed by the advancement of a Corsair Pro catheter.
Movie 3. A Gaia Third guidewire successfully penetrating the proximal true lumen and reaching the aorta in a retrograde manner.
